# Association of abstinence time with semen quality in men who undergoing fertility evaluation: a cross-sectional study from 3052 participants

**DOI:** 10.3389/fendo.2025.1472333

**Published:** 2025-01-24

**Authors:** Guanying Yao, Qinglin Qi, Xianchao Dou, Wen Zhou, Shun Bai, Xi Zhang

**Affiliations:** ^1^ Department of Reproductive Medicine, Nanyang Central Hospital, Nanyang, Henan, China; ^2^ Department of Reproductive Health, Pingxiang Maternal and Child Health Hospital, Pingxiang, Jiangxi, China; ^3^ School of Medical Imaging, Bengbu Medical University, Bengbu, Anhui, China; ^4^ Department of Reproductive Medicine, The Second Affiliated Hospital of Guangzhou University of Chinese Medicine, Guangzhou, Guangdong, China; ^5^ Center for Reproduction and Genetics, The First Affiliated Hospital of USTC, Division of Life Sciences and Medicine, University of Science and Technology of China, Hefei, Anhui, China; ^6^ Department of Obstetrics and Gynecology, Center for Reproductive Medicine, Guangdong Provincial Key Laboratory of Major Obstetric Diseases, Guangdong Provincial Clinical Research Center for Obstetrics and Gynecology, Guangdong-Hong Kong-Macao Greater Bay Area Higher Education Joint Laboratory of Maternal-Fetal Medicine, The Third Affiliated Hospital, Guangzhou Medical University, Guangzhou, Guangdong, China; ^7^ Key Laboratory for Reproductive Medicine of Guangdong Province, The Third Affiliated Hospital, Guangzhou Medical University, Guangzhou, Guangdong, China

**Keywords:** abstinence time, semen quality, semen parameters, DNA fragmentation index, DFI

## Abstract

**Objective:**

Abstinence time has been associated with semen quality, but the results remain controversial.

**Methods:**

This study recruited 3052 men undergoing fertility evaluation. Abstinence time (AT) was categorized as short (0-1 day), WHO-recommended (2-7 days) and long (>7 days). Semen parameters including volume, sperm concentration, progressive motility, total motility, total motility sperm count (TMSC), morphology and DNA fragmentation index were assessed for their association with AT.

**Results:**

Short AT was significantly associated with lower semen volume (P< 0.001), sperm concentration (P= 0.01) and TMSC (P< 0.001), while long AT was significantly associated with higher sperm concentration (P= 0.006), reduced progressive motility (P= 0.005) and total motility (P= 0.02), and higher DFI (P< 0.001). Restricted cubic spline models demonstrated a non-linear relationship between AT and the risk of low semen volume (Pnon-linear < 0.001), sperm concentration (Pnon-linear = 0.039) and TMSC (Pnon-linear < 0.001).

**Conclusions:**

Our findings suggest both short and long AT were significantly associated with lower sperm quality, which indicated the importance of maintaining a recommended AT (2-7days) for semen analysis. Additionally, short abstinence periods may be recommended to maintain optimal sperm DNA integrity.

## Introduction

Human spermatozoa are produced in the seminiferous tubules, release into the rete testis, and subsequently enter the epididymis (1). During transit and storage in the epididymis, immature spermatozoa acquire motility and fertilizing potential, ultimately becoming functionally matured spermatozoa. Several factors correlate with semen quality, one of which is ejaculatory abstinence (2). During periods of ejaculatory abstinence, mature sperm mainly accumulate and greatly expose to reactive oxygen species (ROS) in the epididymis (3, 4). Excessive ROS impair semen quality by reducing sperm motility and causing DNA damage (5). Several studies have indicated that semen volume and sperm count increase, while progressive motility decreases, with longer periods of abstinence (6, 7).

The World Health Organization (WHO) recommends an abstinence period of 2-7 days prior to semen collection for standard analysis. This range is considered optimal for obtaining consistent and reliable sperm parameters, such as sperm count, motility, and morphology, without significantly affecting semen quality. In contrast, the European Society of Human Reproduction and Embryology (ESHRE) suggests a shorter abstinence period of 3-4 days. This recommendation is based on the idea that this specific window may better balance sperm concentration and motility while avoiding potential negative effects, such as reduced sperm motility and increased DNA fragmentation index (DFI), which can occur with longer abstinence periods (8). Current evidence regarding the correlation between short or long abstinence periods and semen quality remains unclear. In this large observational study of 3052 men, we conducted logistic analyses to evaluate the relationship between semen parameters and abstinence time categorized as short (0-1 day), recommend (2-7 days) and long (> 7 days).

## Methods

### Participants

Between April 2020 and May 2022, a total of 3052 men who undergoing fertility evaluation at the first affiliated hospital of University of Science and Technology of China (USTC) were included in this study. Clinical characteristics were collected, and semen quality were assessed for all participants. Exclusion criteria included azoospermia, testicular cancer, cryptorchidism and genetic defects related to the male reproductive tract. This study was approved by the first affiliated hospital of USTC Ethical Committee (No. 2023-RE-196).

### Semen analysis

Semen was collected by masturbation and incubated at 37°C for 30 min for liquefaction. The duration of sexual abstinence was recorded following semen collection. Semen parameters, including sperm concentration, progressive motility and total motility, were assessed using a computer-assisted sperm analysis (CASA) system (SAS-II, SAS Medical, Beijing, China). Sperm morphology was analyzed using Diff-Quick staining (Anke Biotechnology, Hefei, China) under a light microscope (CX33, Olympus Corporation, Tokyo, Japan). Leukocytes were determined by peroxidase staining (Anke Biotechnology, Hefei, China). Antisperm antibodies (AsAs) were detected using the mixed antiglobulin reaction (MAR) test (Anke Biotechnology, Hefei, China). Sperm DFI and high DNA stainability (HDS) were measured using the sperm chromatin structure assay (SCSA, Celula, Chengdu, China).

### Statistical analysis

Qualitative variables were presented as frequencies (percentages), and quantitative variables were expressed as mean ± standard deviation (SD) or median with interquartile range (IQR). Pearson’s chi-square test, Student’s t test and the Mann−Whitney U test were used for comparisons for categorical, parametric and nonparametric data, respectively. Logistic regression was used to examine the association between abstinence time and semen parameters. Restricted cubic spline (RCS) was performed to assess for the dose-response relationships between abstinence time and abnormal semen parameters according to WHO criteria. Covariates initially included age, BMI, smoking, alcohol intake, education, chronic diseases, urogenital infections and varicocele. A P value of less than 0.05 was considered to indicate a statistically significant status. Statistical analyses were performed using GraphPad Prism 9.0 software (San Diego, CA, USA).

## Results

Characteristics of the participants categorized by abstinence time (AT) are shown in **Table 1**. The mean age of the subjects was 30.9 ± 5.0 years, with a mean BMI of 24.7 ± 3.6 kg/m^2^. Among all participants, 1292 (42.3%) smoked tobacco and 1533 (50.2%) consumed alcohol. Educationally, 575 (18.9%) had completed middle school or lower levels, 538 (17.6%) had completed high school, and 1939 (63.5%) had completed college/university. The incidence rates of chronic diseases, urogenital infections and varicocele were 309 (10.1%), 420 (13.8%) and 318 (10.4%), respectively. No significant differences were observed between abstinence time and age, BMI, smoking, drinking, education, chronic diseases, urogenital infections and varicocele.

**Table 1:** Clinical characteristics in men across abstinence time.

Characteristics	Total (n=3052)	Short AT (n=141)	Recommended AT (n=2639)	Long AT (n=272)	*P* value
Clinical parameters					
Age, mean ± s.d.	30.9±5.0	30.6±5.4	30.8±4.9	31.6±5.8	0.06
BMI^a^, mean ± s.d.	24.7±3.6	24.8±3.7	24.7±3.6	24.3±3.7	0.05
Smoking status, n (%)					0.07
Nonsmoker^b^	1760 (57.7)	68 (48.2)	1535 (58.2)	157(57.7)	
Smoker	1292 (42.3)	73 (51.8)	1104 (41.8)	115(42.3)	
Drinking status, n (%)					0.16
Nondrinker^c^	1519 (49.8)	59 (41.8)	1324 (50.2)	136(50.0)	
Drinker	1533 (50.2)	82 (58.2)	1315 (49.8)	136(50.0)	
Education, n (%)					0.38
Middle school or lower^d^	575 (18.9)	27 (19.1)	488 (18.5)	60(22.1)	
High school	538 (17.6)	29 (20.6)	457 (17.3)	52(19.1)	
College/University	1939 (63.5)	85 (60.3)	1694 (64.2)	160(58.8)	
Chronic diseases, n (%)^e^					0.09
No	2743 (89.9)	120 (85.1)	2383 (90.3)	240(88.2)	
Yes	309 (10.1)	21 (14.9)	256 (9.7)	32(11.8)	
Urogenital infections, n (%)^f^					0.10
No	2632 (86.2)	122 (86.5)	2287 (86.7)	223(82.0)	
Yes	420 (13.8)	19 (13.5)	352 (13.3)	49(18.0)	
Varicocele, n (%)					0.43
No	2734 (89.6)	130 (92.2)	2357 (89.3)	247(90.8)	
Yes	318 (10.4)	11 (7.8)	282 (10.7)	25(9.2)	

BMI, body mass index; s.d, standard deviation; AT, abstinence time.

^a^ Calculated as weight in kilograms divided by height in meters squared.

^b^ included never smoking and no smoking during the past 3 months.

^c^ included never drinking and no drinking during the past 3 months.

^d^ included primary school and junior high school.

^e^ included diabetes, hypertension, and hyperlipidemia.

^f^ included epididymitis, prostatitis, balanoposthitis, and seminal vesiculitis.

Compared to men with recommended AT, those with short AT had significantly lower semen volume (*P* < 0.001), sperm concentration (*P* < 0.001) and TMSC (*P* < 0.001), while men with long AT had significantly higher semen volume (*P* < 0.001), sperm concentration (*P* < 0.001), TMSC (*P* < 0.001) and DFI (*P* < 0.001) (**Table 2**). Lower sperm progressive motility and total motility were shown in long AT than those in recommended EA. No significant differences were observed between AT and sperm normal morphology, semen leukocytes, AsAs and HDS. In terms of sperm kinematics, VCL, VSL, VAP, BCF and MAD significantly declined with AT, while ALH, LIN, WOB, and STR significantly increased with AT (**Table 3**).

**Table 2:** Semen parameters in men across abstinence time.

Semen parameters	Total	Short AT (n=141)	Recommended AT (n=2639)	Long AT (n=272)	*P^a^*	*P^b^*	*P^c^*
Volume (ml), median (IQR)	3.1 (2.2-4.2)	2.3 (1.5-2.8)	3.1 (2.2-4.2)	3.9 (2.7-5.0)	<0.001	<0.001	<0.001
Concentration (million/ml), median (IQR)	61.4 (30.8-111.5)	37.4 (18.5-64.0)	60.3 (30.7-109.9)	92.2 (47.7-165.5)	<0.001	<0.001	<0.001
Progressive motility (%), median (IQR)	37.2 (24.1-50.2)	38.4 (22.5-48.8)	37.6 (24.7-50.6)	33.0 (19.9-45.7)	<0.001	0.52	<0.001
Total motility (%), median (IQR)	41.8 (27.8-56.8)	42.8 (28.5-55.3)	42.3 (28.2-57.1)	37.3 (23.2-51.4)	0.003	0.49	<0.001
TMSC (million), median (IQR)	74.6 (25.0-173.2)	29.7 (11.1-59.4)	75.0 (25.7-171.0)	123.4 (35.2-251.2)	<0.001	<0.001	<0.001
Normal morphology (%), median (IQR; n)	6.0 (4.0-8.0; 2790)	6.0 (5.0-8.0; 129)	6.0 (4.0-8.0; 2409)	6.0 (4.0-8.0; 252)	0.38	0.25	0.49
Leukocytes(×10^6^/ml), median (IQR; n)	0.09 (0.03-0.38; 1642)	0.17 (0.05-0.58; 83)	0.08 (0.03-0.38; 1401)	0.09 (0.03-0.34; 158)	0.18	0.07	0.66
AsAs (%), median (IQR; n)	2.0 (1.0-3.0; 1204)	2.0 (1.0-4.0; 64)	2.0 (1.0-3.0; 1021)	1.0 (1.0-3.0; 119)	0.22	0.53	0.12
DFI (%), median (IQR)	13.5 (8.7-20.7; 1207)	11.5 (8.7-16.2; 55)	13.1 (8.5-20.3; 1040)	16.8 (11.8-28.7; 112)	<0.001	0.22	<0.001
HDS (%), median (IQR)	6.9 (5.1-9.6; 1207)	7.0 (5.1-9.8; 55)	7.0 (5.1-9.6; 1040)	6.5 (4.8-8.8; 112)	0.43	0.97	0.19

AT, abstinence time; IQR, interquartile range; TMSC, total motile sperm count; AsAs, antisperm antibodies; DFI, DNA fragmentation index; HDS, high DNA stainability.

*P^a^*: short EA vs Recommended EA vs Long EA; *P^b^*: short EA vs Recommended EA; *P^c^*: Recommended EA vs Long EA.

**Table 3:** Sperm kinematics in men across abstinence time.

Sperm kinematics	Total	Short AT (n=141)	Recommended AT (n=2639)	Long AT (n=272)	*P^a^*	*P^b^*	*P^c^*
VCL (μm/sec), median (IQR)	29.7 (19.8-41.0)	31.5 (20.3-44.2)	30.1 (20.1-41.4)	26.5 (16.8-36.5)	<0.001	0.45	<0.001
VSL (μm/sec), median (IQR)	12.4 (7.6-17.6)	12.6 (7.6-17.8)	12.5 (7.7-17.8)	10.5 (6.1-15.6)	<0.001	0.85	<0.001
VAP (μm/sec), median (IQR)	15.9 (10.0-22.2)	16.7 (10.3-22.9)	16.2 (10.3-22.5)	13.6 (8.4-19.8)	<0.001	0.89	<0.001
BCF (Hz), median (IQR)	7.6 (4.6-10.8)	7.9 (4.6-10.6)	7.7 (4.7-10.9)	6.6 (3.8-9.7)	0.001	0.96	<0.001
ALH (μm/sec), median (IQR)	2.2 (2.0-2.4)	2.2 (1.9-2.4)	2.2 (2.0-2.4)	2.3 (1.9-2.4)	0.04	0.01	0.71
MAD (degrees), median (IQR)	13.1 (9.9-16.1)	13.3 (9.9-15.8)	13.2 (10.0-16.3)	12.1 (8.8-15.2)	0.001	0.77	<0.001
LIN, median (IQR)	38.5 (31.6-43.6)	36.8 (29.3-41.4)	38.5 (31.8-43.7)	38.9 (30.4-43.3)	0.04	0.01	0.59
WOB, median (IQR)	50.6 (44.6-54.8)	49.0 (43.0-53.1)	50.6 (44.8-54.9)	51.0 (43.8-54.7)	0.04	0.01	0.70
STR, median (IQR)	74.1 (61.5-79.1)	71.4 (58.7-77.7)	74.1 (61.6-79.1)	75.3 (60.7-79.2)	0.03	0.01	0.68

AT, abstinence time; VCL, curvilinear velocity; VSL, straight-line velocity; VAP, average path velocity; BCF, frequency of beat cross; ALH, mean amplitude of lateral head displacement; MAD, mean angular displacement; LIN, linearity coefficient; WOB, wobble coefficient; STR, straightness coefficient.

*P^a^*: short EA vs Recommended EA vs Long EA; *P^b^*: short EA vs Recommended EA; *P^c^*: Recommended EA vs Long EA.

As shown in **Table 4**, logistic regression analysis revealed a significantly positive association between short AT and low semen volume (OR = 3.1, 95% CI = 2.0-4.7, *P* < 0.001), low sperm concentration (OR = 1.7, 95% CI = 1.1-2.6, *P* = 0.02) and low TMSC (OR = 2.0, 95% CI = 1.3-3.1, *P* < 0.001). After adjusting for potential confounders including age, BMI, educational levels, smoking, alcohol consumption, chronic diseases, urogenital infections and varicocele), a high risk of poor semen quality was still observed in men with short AT. Additionally, long AT was significantly negatively associated with low sperm concentration (OR = 0.5, 95% CI = 0.3-0.8, *P* = 0.007) and positively associated with low sperm progressive motility (OR = 1.5, 95% CI = 1.2-1.9, *P* = 0.002), low sperm total motility (OR = 1.4, 95% CI = 1.1-1.8, *P* = 0.007) and high sperm DFI (OR = 2.8, 95% CI = 1.7-4.6, *P* < 0.001), and those relationship were still observed after adjusting for several confounders. No significant associations were observed for sperm normal morphology (all *P* > 0.05).

**Table 4:** Odds ratios (95% confidence intervals) for abnormal semen parameters across abstinence time.

Semen parameters	Short AT		Recommended AT		Long AT	
	*OR* (95% CI)	*P*	*OR* (95% CI)	*P*	*OR* (95% CI)	*P*
Crude						
Volume < 1.5 ml	3.1 (2.0-4.7)	< 0.001	Ref		0.6 (0.3-1.0)	0.07
Concentration < 15 million/ml	1.7 (1.1-2.6)	0.02	Ref		0.5 (0.3-0.8)	0.007
Progressive motility <32%	1.1 (0.8-1.6)	0.51	Ref		1.5 (1.2-1.9)	0.002
Total motility <40%	0.9 (0.7-1.3)	0.67	Ref		1.4 (1.1-1.8)	0.007
TMSC < 9 million	2.0 (1.3-3.1)	< 0.001	Ref		0.7 (0.4-1.1)	0.11
Normal morphology < 4%	0.7 (0.4-1.2)	0.22	Ref		1.3 (0.9-1.8)	0.13
DFI > 30%	0.8 (0.2-2.0)	0.69	Ref		2.8 (1.7-4.6)	< 0.001
Adjusted						
Volume < 1.5 ml	3.1 (2.0-4.7)	< 0.001	Ref		0.6 (0.3-1.0)	0.04
Concentration < 15 million/ml	1.8 (1.1-2.7)	0.01	Ref		0.5 (0.3-0.8)	0.006
Progressive motility <32%	1.2 (0.8-1.7)	0.37	Ref		1.4 (1.1-1.9)	0.005
Total motility <40%	1.0 (0.7-1.4)	0.88	Ref		1.4 (1.1-1.8)	0.02
TMSC < 9 million	2.1 (1.4-3.2)	< 0.001	Ref		0.7 (0.4-1.0)	0.08
Normal morphology < 4%	0.7 (0.4-1.3)	0.28	Ref		1.3 (0.9-1.8)	0.17
DFI > 30%	0.9 (0.3-2.3)	0.86	Ref		2.7 (1.6-4.5)	< 0.001

AT, abstinence time; TMSC, total motile sperm count; DFI, DNA fragmentation index; OR, odds ratio; CI, confidence interval.

Adjusted model: adjusted for age, BMI, education, smoking, alcohol consumption, chronic diseases, urogenital infections, and varicocele.

**Figure 1:**
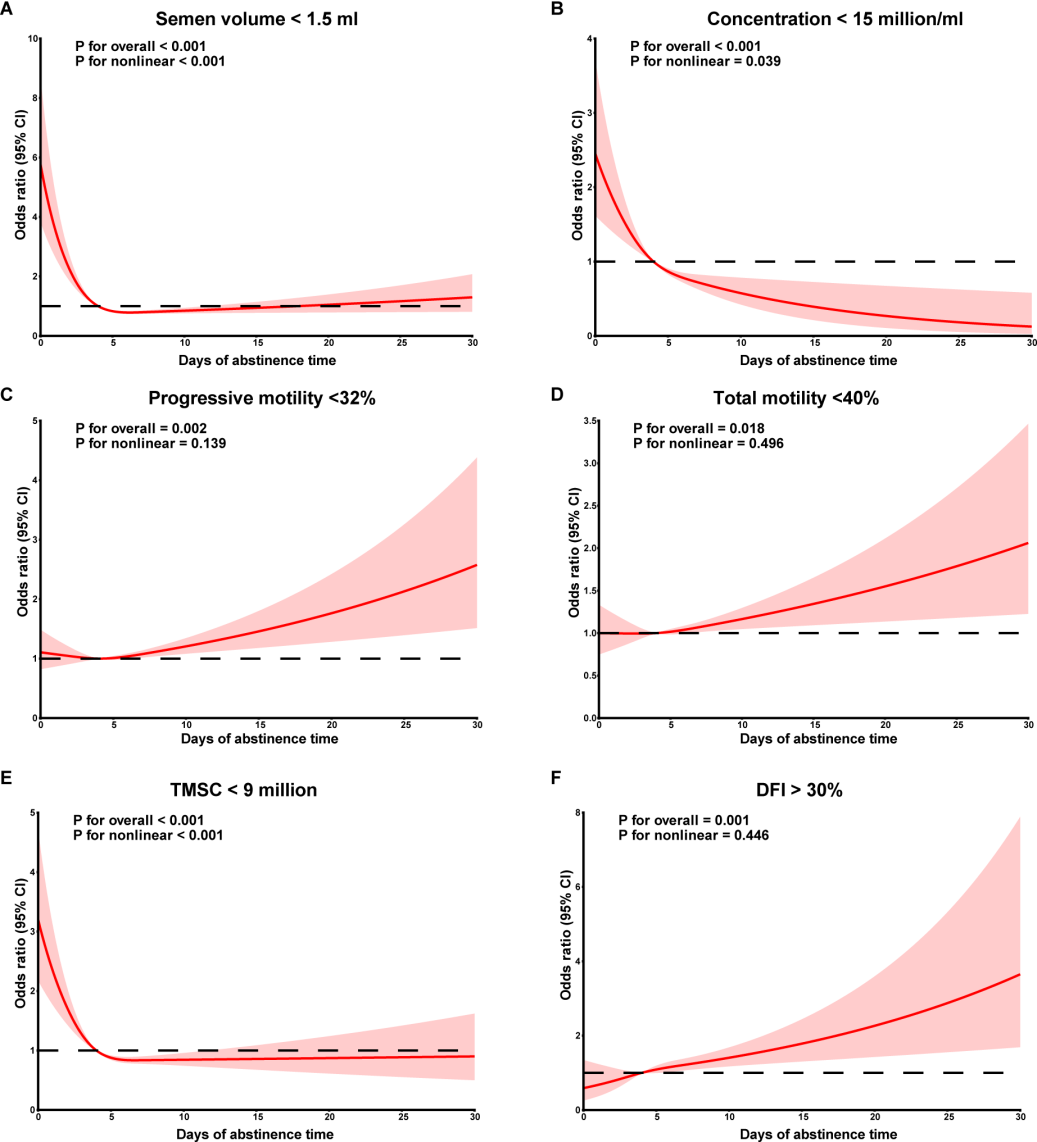
Dose-response curves between abstinence time and semen volume **(A)**, sperm concentration **(B)**, sperm progressive motility **(C)**, sperm total motility **(D)**, TMSC **(E)**, DFI **(F)**. TMSC, total motile sperm count; DFI, DNA fragmentation index.

The dose-response relationships between continuous AT and abnormal semen parameters after adjusting for confounders were further explored using RCS (**Figure 1**). When AT was lower than the median AT in men with recommended AT (4 days), risk of low semen volume (*P* non-linear < 0.001), sperm concentration (*P* non-linear = 0.039) and TMSC (*P* non-linear < 0.001) appeared to decrease with increasing AT, while there was no significant difference for AT longer than 4 days. No significant non-linear relationships were observed between AT and risk of abnormal sperm progressive motility, total motility and sperm DFI.

## Discussion

Semen analysis, including sperm concentration, motility and morphology, has been as the commonly test for evaluating male fertility (9, 10). Several pre-analytical factors, such as time of abstinence and method of semen collection, have been shown to affect semen parameters (11). In this observational study with a relatively large sample size, we assessed the relationship between AT and semen quality in men who undergoing fertility examination. Our results indicate that shorter AT was positively associated with abnormal semen volume, sperm concentration and TMSC, and long AT was positively associated with abnormal sperm progressive motility, total motility and DFI.

The recommended duration of abstinence by the WHO laboratory Manual for the Examination and Processing of Human Semen ranges from 2 to 7 days. The association between AT and semen quality remains controversial (12, 13). Several studies have shown that abstinence duration is significantly linked with semen volume, sperm concentration, motility, normal morphology, and DFI (14–16). However, other studies have not reported significant associations, particularly concerning sperm morphology. Recently, a systematic review suggested that shorter abstinence durations improve semen quality, including lower sperm DFI, higher motility and normal morphology (17). In addition, several guidelines address the beneficial effects of short abstinence duration in reducing sperm DFI (18, 19). However, another review concluded that the effects of short-term abstinence on semen parameters are contradictory and inconclusive (20).

On the other hand, prolonged AT was associated with increased seminal volume, semen leukocytes, sperm concentration, DFI and decreased sperm motility (15). Given that semen volume increases with abstinence duration, the change in sperm count exceeded that in sperm concentration. Recent studies have suggested improvements in sperm count with abstinence periods longer than 5 or 7 days (17, 20), while our study observed TMSC exhibited the most significant changes with increasing AT. As long-term AT appear to reduce sperm motility, TMSC is recommended as a preferred evaluation of semen parameters.

The pathophysiological mechanisms linking abnormal abstinence duration to semen quality remains unclear. Shorter abstinence periods have been reported to affect the duration of sperm retention in the epididymis, which may improve sperm antioxidant capacity and seminal metabolites (21). Prolonged retention of sperm in the epididymis increases susceptibility to damage by ROS (7, 22, 23). ROS at normal physiological levels is essential for the processes of maturation, hyperactivation, capacitation, acrosome reaction, zona pellucida binding and oocyte fusion (24–26). However, an imbalance between ROS and antioxidants leads to oxidative stress, impairing acrosome, mitochondrial, and DNA integrity.

One major strength of our study is its relatively large sample size, which provides high statistical power and precision. The current study also included dose-response relationship between abstinence duration and semen parameters. In addition, we also considered several potential confounding factors, such as age, BMI, drinking, and smoking, that have been previously reported to be associated with semen quality. However, several limitations need to be discussed. First, all semen parameters were obtained from men who undergoing fertility evaluation at a single clinic center and were unable to generalize men with reproductive age. Second, the level of ROS was not assessed in this study, which might affect the relationship between abstinence duration and semen quality. Third, as our study is a retrospective design, potential selection bias is unavoidable. Finally, although we adjusted for several potential confounders, there is still the possibility of residual confounding by unknown factors.

In conclusion, our study showed that both short and long AT were significantly associated with lower sperm quality, which indicated the importance of maintaining a recommended AT for semen analysis. Additionally, short abstinence periods short abstinence periods may be recommended to maintain optimal sperm DNA integrity.

## Data Availability

The original contributions presented in the study are included in the article/supplementary material. Further inquiries can be directed to the corresponding authors.
